# Assessment of risk factors for pelvic organ prolapse using dynamic MRI combined with clinical data

**DOI:** 10.1186/s13244-025-02157-5

**Published:** 2026-03-31

**Authors:** Linghong Qi, Cheng Qian, Jiami Liu, Zhenghua Fei, Yinjian Zhou, Zhi Li

**Affiliations:** 1 Department of Radiology, Huzhou Maternity & Child Health Care Hospital, Huzhou, China; 2 Department of General Surgery Department, Huzhou Maternity & Child Health Care Hospital, Huzhou, China; 3 Department of Gynaecology, Huzhou Maternity & Child Health Care Hospital, Huzhou, China

**Keywords:** Pelvic organ prolapse, Magnetic resonance imaging, Pelvic floor dysfunction, Risk factors

## Abstract

**Objective:**

To investigate the risk factors for pelvic organ prolapse (POP) using dynamic MRI in combination with clinical data.

**Materials and methods:**

Postpartum women who visited between August 2023 and October 2024 were enrolled. Patients were classified into the POP group if the lowest point of the anterior and/or middle compartment organs descended more than 1 cm below the pubococcygeal line (PCL) during straining, and into the non-POP group otherwise. Statistical analysis was conducted to compare MRI parameters and clinical data between the two groups.

**Results:**

A total of 94 cases were assigned to the POP group and 276 to the non-POP group. Univariate and Multivariate logistic regression identified mode of delivery, straining H-line length, H-line difference, and M-line difference as independent risk factors for POP. The cutoff values for straining H-line length, H-line difference, and M-line difference were 5.45 cm, 0.65 cm, and 0.75 cm, respectively. Among these, the H-line difference had the highest area under the curve (AUC = 0.889) and the highest Youden index (0.606). Both H-line difference (r = 0.577, *p* < 0.001) and M-line difference (r = 0.531, *p* < 0.001) showed a positive correlation with the occurrence of POP.

**Conclusion:**

Dynamic MRI combined with clinical data provides an accurate diagnostic approach for POP. Patients with a history of vaginal delivery, straining H-line > 5.45 cm, H-line difference > 0.65 cm, and M-line difference > 0.75 cm should be considered at high risk and may benefit from early clinical intervention.

**Critical relevance statement:**

By integrating clinical data, we aimed to identify risk factors associated with prolapse, thereby providing scientific evidence to support early detection, timely intervention, and preventive strategies for POP in clinical settings.

**Key Points:**

- This study established a comprehensive framework for assessing the risk of pelvic organ prolapse (POP), thereby enhancing diagnostic precision and clinical applicability.
- The H-line difference demonstrated the highest predictive value for POP, with the greatest AUC (0.889) and the highest Youden’s index (0.606).
- H-line and M-line differences were identified as independent risk factors for POP, with cutoff values of 0.65 cm and 0.75 cm.

**Graphical Abstract:**

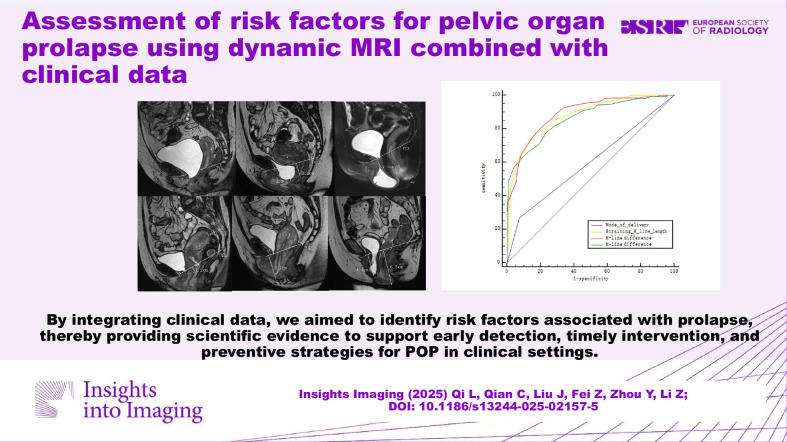

## Introduction

Pelvic organ prolapse (POP) is a condition characterized by the descent of pelvic and female reproductive organs, resulting in abnormal anatomical positioning or function. It is primarily caused by injury or weakening of the pelvic floor muscles and connective tissues [1]. POP can exert excessive traction on the uterine ligaments, leading to pelvic congestion and lower back pain. In severe cases, the prolapsed uterus and vaginal wall may be chronically exposed and subject to friction against clothing, resulting in erosion, ulceration, bleeding, infection, and, in some instances, purulent discharge, foul odor, or even malignant transformation [2]. The prevalence of genital prolapse varies greatly according to various studies. This variability is due to differences in the designs of the studies conducted, as well as the use of different approaches to the diagnosis of the conditions under study. In studies based on objective gynecological examination without considering subjective symptoms in the examined patients, the prevalence of pelvic organ prolapse (POP) reaches 50% [3]. With an aging population, the number of women seeking medical care for POP is projected to increase by 35% over the next decade [4]. By 2050, an estimated 9.2 million women in the United States alone will be impacted by POP [5], imposing a substantial burden on individuals, healthcare systems, and socioeconomic structures. The diagnosis of POP primarily relies on patient-reported symptoms, physical examination, and auxiliary imaging. Magnetic resonance imaging (MRI), due to its high soft-tissue resolution, lack of ionizing radiation, and ability to visualize the entire pelvis and pelvic floor support structures, has been widely adopted for the evaluation of pelvic floor dysfunction [6]. By assessing the displacement of pelvic organs and structural alterations in multiple planes and dimensions during rest and straining (Valsalva maneuver), MRI enables comprehensive functional evaluation of the pelvic floor [7, 8] and has become the preferred imaging modality for patients with POP [9]. In this study, dynamic MRI was utilized to assess dimensional changes in the anterior and middle pelvic compartments in postpartum women. By integrating clinical data, we aimed to identify risk factors associated with prolapse, thereby providing scientific evidence to support early detection, timely intervention, and preventive strategies for POP in clinical settings.

## Materials and methods

### General information

Postpartum women who visited Huzhou Maternity & Child Health Care Hospital between August 2023 and October 2024 were enrolled in this retrospective study. All participants were 6–8 weeks postpartum at the time of enrollment. Inclusion criteria included maternal age > 18 years and the ability to perform the Valsalva maneuver adequately. Exclusion criteria were a history of pelvic surgery or contraindications to MRI. This study was approved by the Ethics Committee of Huzhou Maternity & Child Health Care Hospital (IRM No.: 2025-J-029).

### Imaging protocol

All MRI scans were performed using a Siemens Avanto 1.5-T scanner with an abdominal coil. Axial images were acquired with a slice thickness of 2 mm, an interslice gap of 0.3 mm, and a field of view of 380 × 380 mm. Sagittal and coronal images were obtained with a slice thickness of 5 mm, an interslice gap of 1.5 mm, and two signal excitations. Axial TSE-T1WI: TR = 995 ms, TE = 12 ms, matrix = 205 × 256. Axial TSE-T2WI: TR = 7260 ms, TE = 72 ms, matrix = 192 × 256. Sagittal TSE-T1WI: TR = 797 ms, TE = 12 ms, matrix = 205 × 256. Sagittal TSE-T2WI: TR = 4271 ms, TE = 69 ms, matrix = 240 × 320. Coronal TSE-T2WI: TR = 4300 ms, TE = 69 ms, matrix = 240 × 320. Participants were instructed to refrain from urinating for 2 h prior to scanning to ensure moderate bladder filling. They were also trained to alternate between resting and straining (Valsalva) maneuvers. During imaging, participants were positioned supine with a cushion placed under the knees to simulate the lithotomy position.

### Measurement methods of pelvic floor parameters

The pubococcygeal line (PCL) is defined as a straight line connecting the inferior border of the pubic symphysis to the last coccygeal joint on the midsagittal plane. It serves as the primary reference line for assessing POP and represents the pelvic floor level [10, 11]. The reference point for the anterior compartment is the lowest point of the bladder base, while for the middle compartment, it is the lowest point of the anterior cervical lip (or the vaginal vault in post-hysterectomy patients).

**Fig. 1:**
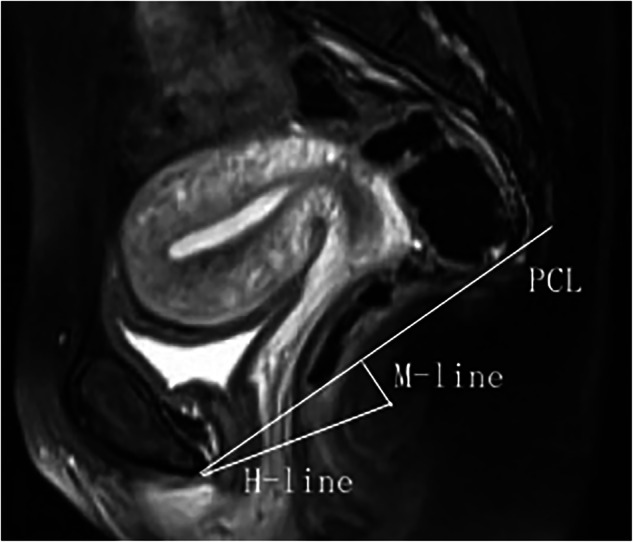
Measurement of the PCL, H-line, and H-line diagram on the median sagittal T2-weighted image

The H line represents the anteroposterior diameter of the levator hiatus, measured from the inferior border of the pubic symphysis to the posterior wall of the anorectal junction. The M line indicates pelvic floor descent and is defined as the perpendicular distance from the end of the H line to the PCL [12, 13] (Fig. 1). Differences in the H- and M-lines are calculated as the measurement during the straining phase minus that during the resting phase.

**Fig. 2:**
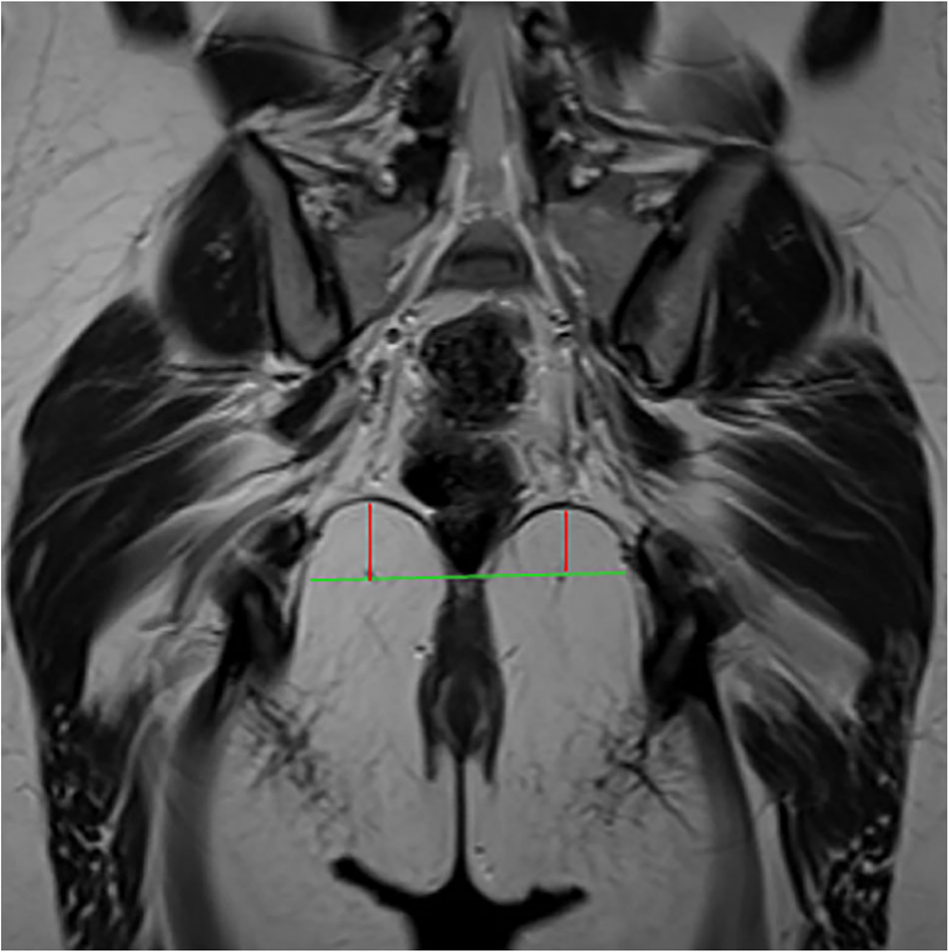
Measurement of the iliococcygeus muscle height on the coronal plane at the mid-anal canal. The green line represents the horizontal plane corresponding to the medial attachment point of the iliococcygeus muscle, while the red line indicates the vertical height from this reference point to the lowest point of the iliococcygeus muscle

The thickness of the puborectalis muscle was measured at its anterior one-third on the axial plane at the level of the inferior border of the pubic symphysis. The anteroposterior diameter, transverse diameter, and area of the levator hiatus were also measured on the same axial plane, with all measurements taken along the inner edge [14]. The thickness of the iliococcygeus muscle was measured at the mid-level of the anal canal on the coronal plane [14]. The height of the iliococcygeus muscle was defined as the vertical distance from the inferior margin of its highest point to the level of its medial attachment (Fig. 2).

### POP grading criteria

**Fig. 3:**
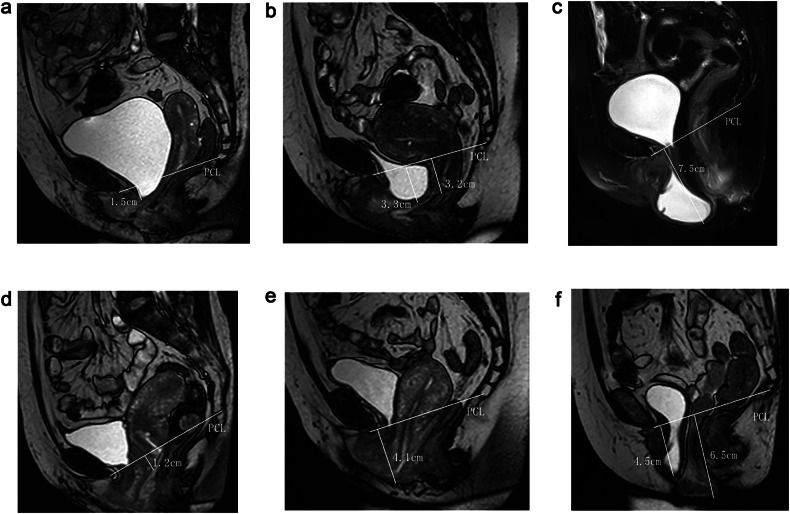
Schematic of POP classification. All images are median sagittal T2-weighted images during the straining phase, measuring the vertical distance from the lowest edge of the pelvic organ to the PCL line. **a** Mild bladder prolapse; **b** moderate bladder and uterine prolapse; **c** severe bladder prolapse; **d** mild uterine prolapse; **e** moderate uterine prolapse; **f** severe uterine prolapse with moderate bladder prolapse

The grading of anterior and middle compartment POP was evaluated based on the vertical distance of the reference points below the PCL during the straining phase [15], as presented in Table 1 and illustrated in Fig. 3.

**Table 1:** POP grading criteria

Grading	Mild prolapse	Moderate prolapse	Severe prolapse
Criteria	1–3 cm	3–6 cm	> 6 cm

### Evaluation of levator ani muscle injury and edema

Levator ani muscle injury was assessed based on the criteria established by DeLancey et al [16]. The total score from both sides was used to classify injury severity: no injury, mild injury, and severe injury. A unilateral complete tear was also classified as severe. Edema of the levator ani was assessed using a similar scale: 0 points, no unilateral edema; 1 point, edema area < 1/2; 2 points, edema area ≥1/2; 3 points, complete edema. The total bilateral score was used to classify the degree of edema as none (0 points), mild (1–3 points), or severe (4–6 points). Unilateral complete edema was classified as severe.

### Statistical analysis

Statistical analysis was performed using SPSS version 22.0 and MedCalc version 15.2 (Chinese edition). Continuous variables with a normal distribution were expressed as mean ± standard deviation (x̄ ± s), and comparisons between groups were conducted using the independent samples *t*-test. Non-normally distributed data were presented as medians with interquartile ranges [M(P25–P75)] and analyzed using non-parametric tests. Categorical data were expressed as frequencies or percentages and compared using the chi-square (χ²) test. Pearson correlation analysis was used to assess correlations between two variables. Variables with statistical significance in univariate analysis were further analyzed using multivariate logistic regression. Receiver operating characteristic (ROC) curves were plotted, and the area under the curve (AUC), sensitivity, and specificity were calculated. A *p*-value < 0.05 was considered statistically significant.

## Results

### Comparison of general characteristics

In this study, postpartum women were categorized into the POP group if the lowest margin of the anterior and/or middle compartment organs during the straining phase on MRI was located more than 1 cm below the PCL; otherwise, they were classified into the non-POP group. A total of 94 patients were included in the POP group and 276 in the non-POP group. In the non-POP group, 36 patients reported a sensation of perineal heaviness, 25 experienced difficulty in urination or defecation, and 215 were asymptomatic. In the POP group, 25 patients reported perineal heaviness, 12 experienced urinary or defecatory difficulties, 3 presented with vaginal or bladder prolapse, and 54 were asymptomatic. Among patients with POP, 35 had mild prolapse, 56 had moderate prolapse, and 3 had severe prolapse. Univariate analysis revealed statistically significant differences between the two groups in terms of mode of delivery, infant birth weight, straining H-line length, H-line difference, resting M-line length, straining M-line length, M-line difference, and resting levator hiatus area (*p* < 0.05) (Table 2).

**Table 2:** Comparison of general information between the two groups of patients

	Non-POP group (*N* = 276)	POP group (*N* = 94)	Statistical value	*p*-value
Age, years	30.36 ± 4.42	31.59 ± 4.59	2.301	0.022
BMI	25.66 ± 3.70	25.99 ± 4.19	0.712	0.477
Number of childbirths	1.43 ± 0.56	1.48 ± 0.56	0.712	0.477
Mode of delivery				
Caesarean birth	74	6	17.267	< 0.001
Vaginal birth	202	88		
Singleton fetus	267	91	0.001	0.974
Multiple birth	9	3		
Baby birth weight, kg	3.35 ± 0.41	3.46 ± 0.34	2.409	0.016
Resting H-line length	4.70 ± 0.70	4.85 ± 0.70	1.780	0.077
Straining H-line length	4.91 ± 0.71	6.13 ± 0.80	13.875	< 0.001
H-line difference	0.30 (−0.10 to 0.60)	1.20 (0.70–1.60)	11.298	< 0.001
Resting M-line length	1.05 ± 0.38	1.15 ± 0.40	2.076	0.039
Straining M-line length	1.39 ± 0.68	2.46 ± 0.77	12.667	< 0.001
M-line difference	0.40 (0.10–0.70)	1.10 (0.80–1.70)	10.517	< 0.001
Thickness of the right puborectalis muscle	0.30 (0.30–0.47)	0.30 (0.30–0.45)	0.530	0.596
Thickness of the left puborectalis muscle	0.31 (0.30–0.45)	0.30 (0.30–0.40)	1.079	0.281
Puborectalis muscle injury				
No injury	166	54	1.400	0.496
Mild injury	98	33		
Severe injury	12	7		
Puborectalis muscle edema				
No edema	234	71	1.400	0.496
Mild edema	39	21		
Severe edema	3	2		
Thickness of the right iliopsoas muscle	0.28 ± 0.09	0.31 ± 0.09	1.987	0.048
Thickness of the left iliopsoas muscle	0.30 ± 0.09	0.32 ± 0.09	2.114	0.035
Injury to the iliopsoas muscle				
No injury	145	53	0.963	0.618
Mild injury	116	38		
Severe injury	15	3		
Edema of the iliopsoas muscle				
No edema	242	87	2.013	0.365
Mild edema	32	7		
Severe edema	2	0		
Height of the right iliopsoas muscle	1.93 ± 0.49	2.04 ± 0.53	1.813	0.071
Height of the left iliopsoas muscle	1.98 ± 0.50	2.07 ± 0.54	1.554	0.121
Anterior posterior/transverse diameter ratio of the levator hiatus	1.53 ± 0.30	1.50 ± 0.31	1.041	0.298
Area of the levator hiatus	14.00 (1.78–18.00)	17.30 (2.10–21.00)	3.092	0.002

### Multivariate analysis of POP in postpartum women

Variables with *p* < 0.05 in the univariate analysis were included in a logistic regression model. The presence or absence of POP was set as the dependent variable, while mode of delivery, infant birth weight, straining H-line length, H-line difference, resting M-line length, straining M-line length, M-line difference, and resting levator hiatus area were included as independent variables. Multivariate logistic regression analysis indicated that mode of delivery, straining H-line length, H-line difference, and M-line difference were independent risk factors for POP in postpartum women (Table 3).

**Table 3:** Multivariate analysis of POP in postpartum women

Variable	B value	Standard deviation	Wald *X*² value	OR (95% CI)	*p*-value
Mode of delivery	1.242	0.623	3.976	3.462 (1.021–11.732)	0.046
Straining H-line length	1.466	0.368	15.873	4.331 (2.106–8.906)	< 0.001
H-line difference	1.767	0.436	16.458	5.855 (2.493–13.750)	< 0.001
M-line difference	1.309	0.631	4.305	3.701 (1.075–12.742)	0.038

### ROC curves, sensitivity, and specificity of independent risk factors

**Fig. 4:**
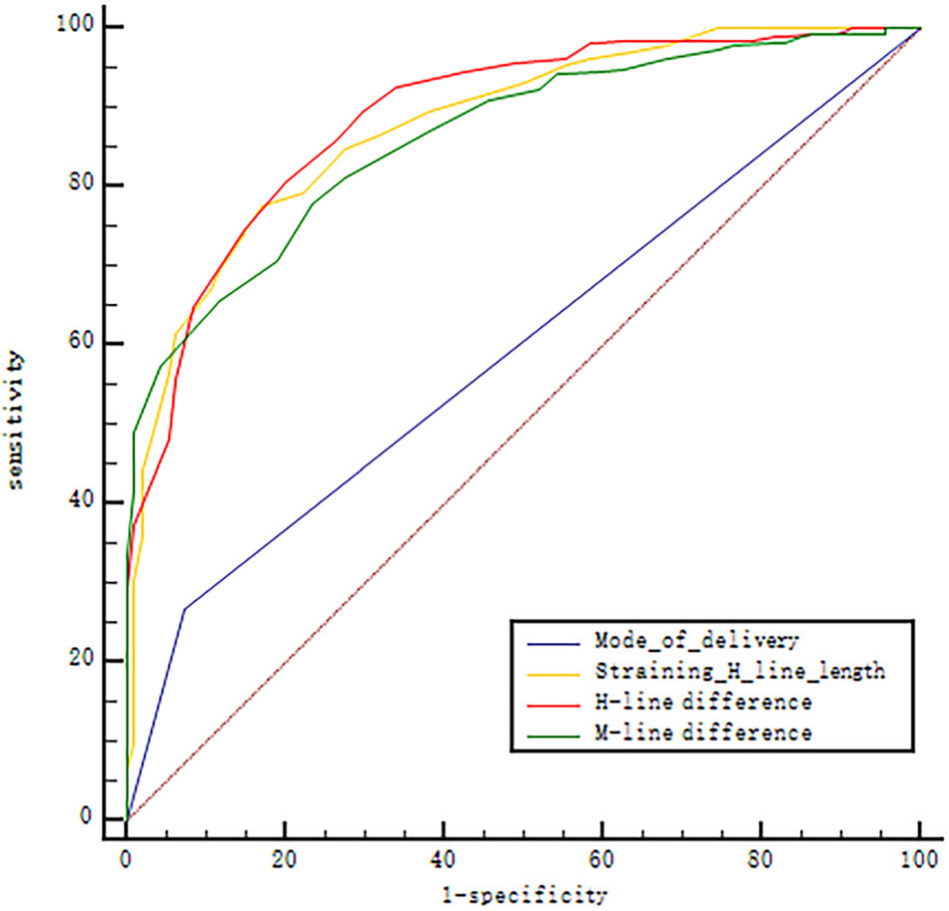
ROC curve assessing the predictive performance of delivery mode, straining H-line length, H-line difference, and M-line difference for postpartum POP

Among the independent risk factors, the H-line difference exhibited the largest AUC of 0.889 (Fig. 4). The highest Youden index was also observed for the H-line difference, with a value of 0.606 (Table 4).

**Table 4:** ROC curves, sensitivity, and specificity of independent risk factors

Variable	AUC	Sensitivity	Specificity	Youden index	Cutoff
Mode of delivery	0.597	92.6%	26.8%	0.194	-
Straining H-line length	0.877	83.0%	77.5%	0.605	5.45
H-line difference	0.889	79.8%	80.8%	0.606	0.65
M-line difference	0.863	76.6%	77.9%	0.545	0.75

### Linear correlation analysis between H line difference, M line difference, and the occurrence of POP

A significant positive correlation was observed between the H-line difference and the occurrence of POP (r = 0.577, *p* < 0.001). Similarly, the M-line difference was also positively correlated with the occurrence of POP (r = 0.531, *p* < 0.001).

## Discussion

The levator ani muscle plays a critical role in supporting the pelvic floor and maintaining the anatomical positions of the vagina, rectum, and urethra. Vaginal delivery is a physiologically demanding process during which the fetal head excessively stretches and dilates the pelvic musculature [17]. This process can also compress the pudendal nerve, impairing its innervation and leading to muscle fiber atrophy and thinning [18], which in turn reduces muscle strength and endurance [19]. These changes compromise pelvic support and contribute to the development of postpartum POP. Epidemiological data suggest that approximately 50% of postpartum women experience varying degrees of POP [20]. In contrast, the incidence of POP in this study was 25.4% (94/370), which is markedly lower than previously reported. This difference may be attributed to the stricter diagnostic criteria employed in the current study, in which POP was defined only when the lowest point of the anterior and/or middle compartment descended more than 1 cm below the PCL during straining. Cases that did not meet this threshold were categorized as non-POP. Previous research has consistently identified vaginal delivery as a major risk factor for POP [21], and our findings support this association. In this study, vaginal delivery was found to be an independent risk factor for POP, with a sensitivity of 92.6%. However, its specificity and Youden index were relatively low, indicating limited discriminative power. Further studies with larger sample sizes are warranted to improve the predictive accuracy and refine risk stratification for POP occurrence. The risk of POP has been shown to increase with advancing age [22]. In the present study, although univariate analysis revealed a statistically significant difference in age, age was not identified as an independent risk factor for POP. This finding may be attributed to the fact that all participants were postpartum women, resulting in a relatively homogeneous age distribution.

Clinicians commonly use the Pelvic Organ Prolapse Quantification (POP-Q) system to diagnose POP [23]. However, this method is technically demanding and difficult to implement in primary healthcare settings [24]. Previous studies have demonstrated that MRI findings of anterior and middle compartment POP correlate well with POP-Q grading [25, 26]. Therefore, in this study, patients were categorized according to MRI findings, which offer a simpler and more intuitive means of assessment. The use of MRI in the field of pelvic floor assessment has become increasingly widespread and is now considered the gold standard for visualizing pelvic floor muscles. MRI provides detailed insights into the anatomical relationships of the levator ani muscle and pelvic organs from multiple perspectives, including morphology, microstructure, and biomechanics. Compared to ultrasound, MRI is more effective in detecting hidden damage to pelvic support structures and offers a comprehensive evaluation of pelvic floor structure and function [27]. Additionally, MRI measurements exhibit excellent reproducibility and consistency [28]. The Valsalva maneuver involves the patient taking a deep breath, holding it, and then exerting downward pressure. This action helps assess specific changes in the pelvic region by compressing and stretching the pelvic floor muscles, thereby increasing the pelvic load. As a result, the fibers of the levator ani muscle are passively pulled and expanded, leading to an increase in the anteroposterior diameter and area of the levator ani hiatus. Combining MRI with the Valsalva maneuver allows for a comprehensive assessment of both the anatomical and functional characteristics of the pelvic floor muscles and pelvic organs [29–31].

The H-line and M-line are used in MRI to assess the size of the levator ani hiatus [32]. The H-line reflects the anteroposterior diameter of the hiatus, while the M-line reflects the degree of pelvic floor descent. During the resting phase, the length of the H-line does not exceed 5 cm [33], and the length of the M-line does not exceed 2 cm [32, 33]. During the Valsalva maneuver, both H-line and M-line lengths may slightly increase. In this study, the mean resting-phase H-line length in the POP group was 4.85 cm, which was greater than the mean of 4.70 cm in the non-POP group, though the difference was not statistically significant. However, the mean H-line length during the Valsalva maneuver in the POP group was 6.13 cm, significantly greater than the non-POP group’s mean of 4.91 cm (*p* < 0.001). The mean resting-phase M-line length in the POP group was 1.15 cm, which was greater than the non-POP group’s average of 1.05 cm (*p* = 0.039). During the Valsalva maneuver, the POP group had a mean M-line length of 2.46 cm, which was significantly greater than the non-POP group’s average of 1.39 cm (*p* < 0.001).In this study, we also calculated the differences in H-line and M-line lengths between the resting and straining phases and performed statistical analysis. The results showed that both H-line and M-line differences are independent risk factors for POP, with cutoff values of 0.65 cm and 0.75 cm, respectively. Moreover, both were positively correlated with the occurrence of POP. Although the straining H-line length was also an independent risk factor for POP, its AUC, specificity, and Youden index were all lower than those of the H-line difference. Overall, the H-line difference demonstrated superior diagnostic performance for POP compared to the straining H-line length. In this study, the mean resting H-line length in both groups did not exceed the 5 cm reported in the literature, and the mean M-line length did not exceed the 2 cm reported in previous studies. This may be due to the study population consisting of young postpartum women. Previous reports have indicated that with increasing age, the levator ani hiatus enlarges [34], which may result in an increase in both the H-line and M-line measurements. The larger the levator ani hiatus area, the greater the likelihood of POP occurrence [35], a finding consistent with previous research [36–38]. In this study, the average levator ani hiatus area in the POP group was 17.30 cm², which was significantly larger than the average of 14.0 cm² in the non-POP group (*p* = 0.002). However, multivariate analysis revealed that the area of the levator ani hiatus was not an independent risk factor for POP. This may be because the control group in this study was not composed of healthy individuals. Future studies should include a healthy control group to further verify the relationship between the levator ani hiatus and POP occurrence.

However, this study has several limitations. First, this study was conducted at a single center and employed a retrospective design, which may introduce certain biases. Future research should involve multiple centers to enhance the accuracy of multivariate logistic regression analyses, develop a predictive model for POP occurrence, and validate it using external datasets. Second, as a cross-sectional study, longitudinal follow-up was not performed, limiting the ability to assess the dynamic progression of POP recovery. Addressing this limitation will be a focus of future investigations.

In conclusion, dynamic MRI combined with clinical data offers an accurate diagnostic tool for POP, especially in cases with a history of vaginal delivery, a straining H-line length > 5.45 cm, an H-line difference > 0.65 cm, and an M-line difference > 0.75 cm. Clinicians should consider timely intervention in such cases.

## Data Availability

The datasets generated or analyzed during the study are available from the corresponding author on reasonable request.

## Abbreviations

AUC: Area under the curve
MRI: Magnetic resonance imaging
PCL: Pubococcygeal line
POP: Pelvic organ prolapse
ROC: Receiver operating characteristic

## References

[1] Zhang C, Li X, Xie B et al (2025) A novel pelvic magnetic resonance imaging measurement for pelvic organ prolapse evaluation. Am J Obstet Gynecol 232:383.e381–383.e388 10.1016/j.ajog.2024.10.007 39426783

[2] Resende APM, Bernardes BT, Stüpp L et al (2019) Pelvic floor muscle training is better than hypopressive exercises in pelvic organ prolapse treatment: an assessor-blinded randomized controlled trial. Neurourol Urodyn 38:171–179 30311680 10.1002/nau.23819

[3] Donaldson K, Huntington A, De Vita R (2021) Mechanics of uterosacral ligaments: current knowledge, existing gaps, and future directions. Ann Biomed Eng 49:1788–1804 33754254 10.1007/s10439-021-02755-6

[4] Brown HW, Hegde A, Huebner M et al (2022) International urogynecology consultation chapter 1 committee 2: epidemiology of pelvic organ prolapse: prevalence, incidence, natural history, and service needs. Int Urogynecol J 33:173–187 34977950 10.1007/s00192-021-05018-z

[5] Wu JM, Hundley AF, Fulton RG, Myers ER (2009) Forecasting the prevalence of pelvic floor disorders in U.S. women: 2010 to 2050. Obstet Gynecol 114:1278–1283 19935030 10.1097/AOG.0b013e3181c2ce96

[6] Welch EK, Ross W, Dengler KL, Gruber DD, Lamb S (2024) The “ins and outs” of dynamic magnetic resonance imaging for female pelvic organ prolapse. Int Urogynecol J 35:2223–2225 39340644 10.1007/s00192-024-05935-9 PMC11638283

[7] Strauss C, Lienemann A, Spelsberg F, Bauer M, Jonat W, Strauss A (2012) Biomechanics of the female pelvic floor: a prospective trail of the alteration of force-displacement-vectors in parous and nulliparous women. Arch Gynecol Obstet 285:741–747 21879335 10.1007/s00404-011-2024-5

[8] Bitti GT, Argiolas GM, Ballicu N et al (2014) Pelvic floor failure: MR imaging evaluation of anatomic and functional abnormalities. Radiographics 34:429–448 24617690 10.1148/rg.342125050

[9] Salvador JC, Coutinho MP, Venâncio JM, Viamonte B (2019) Dynamic magnetic resonance imaging of the female pelvic floor—a pictorial review. Insights Imaging 10:4 30689115 10.1186/s13244-019-0687-9 PMC6352388

[10] Rosenkrantz AB, Lewis MT, Yalamanchili S, Lim RP, Wong S, Bennett GL (2014) Prevalence of pelvic organ prolapse detected at dynamic MRI in women without history of pelvic floor dysfunction: comparison of two reference lines. Clin Radiol 69:e71–e77 24290773 10.1016/j.crad.2013.09.015

[11] García del Salto L, de Miguel Criado J, Aguilera del Hoyo LF et al (2014) MR imaging-based assessment of the female pelvic floor. Radiographics 34:1417–1439 25208288 10.1148/rg.345140137

[12] Colaiacomo MC, Masselli G, Polettini E et al (2009) Dynamic MR imaging of the pelvic floor: a pictorial review. Radiographics 29:e35 19270071 10.1148/rg.e35

[13] Fielding JR (2002) Practical MR imaging of female pelvic floor weakness. Radiographics 22:295–304 11896220 10.1148/radiographics.22.2.g02mr25295

[14] Cai XR, Qiu L, Wu HJ, Liu SR (2013) Assessment of levator ani morphology and function in asymptomatic nulliparous women via static and dynamic magnetic resonance imaging. Int J Gynaecol Obstet 121:233–239 23518136 10.1016/j.ijgo.2013.01.022

[15] Woodfield CA, Krishnamoorthy S, Hampton BS, Brody JM (2010) Imaging pelvic floor disorders: trend toward comprehensive MRI. AJR Am J Roentgenol 194:1640–1649 20489108 10.2214/AJR.09.3670

[16] DeLancey JO, Morgan DM, Fenner DE et al (2007) Comparison of levator ani muscle defects and function in women with and without pelvic organ prolapse. Obstet Gynecol 109:295–302 17267827 10.1097/01.AOG.0000250901.57095.ba

[17] Ashton-Miller JA, Delancey JO (2009) On the biomechanics of vaginal birth and common sequelae. Annu Rev Biomed Eng 11:163–176 19591614 10.1146/annurev-bioeng-061008-124823 PMC2897058

[18] South MM, Stinnett SS, Sanders DB, Weidner AC (2009) Levator ani denervation and reinnervation 6 months after childbirth. Am J Obstet Gynecol 200:519.e511–517 10.1016/j.ajog.2008.12.044 19268880

[19] Hilde G, Staer-Jensen J, Siafarikas F, Gjestland K, Ellström Engh M, Bø K (2013) How well can pelvic floor muscles with major defects contract? A cross-sectional comparative study 6 weeks after delivery using transperineal 3D/4D ultrasound and manometer. BJOG 120:1423–1429 23834432 10.1111/1471-0528.12321

[20] Nemeth Z, Ott J (2011) Complete recovery of severe postpartum genital prolapse after conservative treatment—a case report. Int Urogynecol J 22:1467–1469 21614441 10.1007/s00192-011-1452-x

[21] Blomquist JL, Muñoz A, Carroll M, Handa VL (2018) Association of delivery mode with pelvic floor disorders after childbirth. JAMA 320:2438–2447 30561480 10.1001/jama.2018.18315 PMC6583632

[22] Vergeldt TF, Weemhoff M, IntHout J, Kluivers KB (2015) Risk factors for pelvic organ prolapse and its recurrence: a systematic review. Int Urogynecol J 26:1559–7324 25966804 10.1007/s00192-015-2695-8 PMC4611001

[23] Treszezamsky AD, Filmar G, Panagopoulos G, Vardy MD, Ascher-Walsh CJ (2012) Teaching of pelvic organ prolapse quantification system among obstetrics/gynecology and urology residents in the United States. Female Pelvic Med Reconstr Surg 18:37–40 10.1097/SPV.0b013e318241f7f2 22453266

[24] Wang YT, Jiang JY, Han JS (2016) A review of the pelvic organ prolapse quantification system in China. Int Urogynecol J 27:287–290 10.1007/s00192-015-2830-6 26353847

[25] Swamy N, Bajaj G, Olliphant SS et al (2021) Pelvic floor imaging with MR defecography: correlation with gynecologic pelvic organ prolapse quantification. Abdom Radiol (NY) 46:1381–1389 32211947 10.1007/s00261-020-02476-9

[26] Jha P, Sarawagi R, Malik R, Kumar A, Pushpalatha K (2023) Static and dynamic magnetic resonance imaging in female pelvic floor dysfunction: correlation with pelvic organ prolapse quantification. Cureus 15:e44915 10.7759/cureus.44915 PMC10560544 37814774

[27] El Sayed RF, Alt CD, Maccioni F et al (2017) Magnetic resonance imaging of pelvic floor dysfunction—joint recommendations of the ESUR and ESGAR Pelvic Floor Working Group. Eur Radiol 27:2067–2085 27488850 10.1007/s00330-016-4471-7 PMC5374191

[28] Alt CD, Hampel F, Hallscheidt P, Sohn C, Schlehe B, Brocker KA (2016) 3 T MRI-based measurements for the integrity of the female pelvic floor in 25 healthy nulliparous women. Neurourol Urodyn 35:218–223 25393071 10.1002/nau.22697

[29] Yang J, Zhang K, Han J, Wang Y, Yao Y, Zhou Y (2023) Comparison of the anterior pelvis and levator ani muscle on MRI in women with and without anterior pelvic organ prolapse. Int Urogynecol J 34:1885–1890 36786852 10.1007/s00192-023-05464-x

[30] Neshatian L, Lam JP, Gurland BH, Liang T, Becker L, Sheth VR (2022) MRI biomarker of muscle composition is associated with severity of pelvic organ prolapse. Tech Coloproctol 26:725–733 35727428 10.1007/s10151-022-02651-8

[31] Flusberg M, Kobi M, Bahrami S et al (2021) Multimodality imaging of pelvic floor anatomy. Abdom Radiol (NY) 46:1302–1311 31555847 10.1007/s00261-019-02235-5

[32] Gilyadova A, Ishchenko A, Puchkova E et al (2023) Diagnostic value of dynamic magnetic resonance imaging (dMRI) of the pelvic floor in genital prolapses. Biomedicines 11:2849 10.3390/biomedicines11102849 PMC10604435 37893222

[33] Clark NA, Brincat CA, Yousuf AA, Delancey JO (2010) Levator defects affect perineal position independently of prolapse status. Am J Obstet Gynecol 203:595.e517–522 10.1016/j.ajog.2010.07.044 PMC3360540 20869037

[34] Cantiani C, Sgamma D, Grossi E et al (2020) Posterior pelvic tilt is a risk factor for rectal prolapse: a propensity score matching analysis. Tech Coloproctol 24:46346–46349 10.1007/s10151-020-02179-9 32170509

[35] Van Geelen H, Ostergard D, Sand P (2018) A review of the impact of pregnancy and childbirth on pelvic floor function as assessed by objective measurement techniques. Int Urogynecol J 29:327–338 29332252 10.1007/s00192-017-3540-z

[36] Handa VL, Roem J, Blomquist JL, Dietz HP, Muñoz A (2019) Pelvic organ prolapse as a function of levator ani avulsion, hiatus size, and strength. Am J Obstet Gynecol 221:41.e1–41.e7 10.1016/j.ajog.2019.03.004 PMC6592735 30885773

[37] Siahkal SF, Iravani M, Mohaghegh Z, Sharifipour F, Zahedian M, Nasab MB (2021) Investigating the association of the dimensions of genital hiatus and levator hiatus with pelvic organ prolapse: a systematic review. Int Urogynecol J 32:2095–210938 33523259 10.1007/s00192-020-04639-0

[38] Dietz HP (2019) Ultrasound in the assessment of pelvic organ prolapse. Best Pract Res Clin Obstet Gynaecol 54:12–30 30082146 10.1016/j.bpobgyn.2018.06.006

